# Rare Root Canal Configuration in a Second Maxillary Molar: A Case Report

**DOI:** 10.7759/cureus.80061

**Published:** 2025-03-04

**Authors:** Denitsa Zaneva-Hristova, Tsvetelina Borisova-Papancheva

**Affiliations:** 1 Department of Conservative Dentistry and Endodontics, The Faculty of Dental Medicine-Varna, Varna, BGR; 2 Department of Conservative Dentistry and Oral Pathology, Medical University of Varna, Varna, BGR

**Keywords:** c shape, maxillary second molar, one root canal, root canal anatomy, single root, vertucci classification

## Abstract

Endodontic treatment is a routine procedure performed daily in dental practices. It requires a good knowledge of all the variations in the configurations of the root canal systems of different groups of teeth. A thorough analysis of all available imaging studies is necessary. Difficulties may arise in the analysis and treatment protocol for rare morphological features of the root canal system. A representative of these cases is the single-rooted upper second molar with one wide canal.

The purpose of this article is to present a case with an unusual upper second molar morphology: a single wide root canal. Magnification and good anatomical understanding facilitated detection and treatment, preserving the tooth's function within the dentition.

## Introduction

The main goal of endodontic treatment is the removal of infected tissues, disinfection of the root canal system, and prevention of re-infection, which is achieved by three-dimensional obturation of the root canal. To achieve all these requirements, one needs to know good morphological features, as well as all configurations in the root canal system. The most common configuration in the upper second molar is the presence of three roots, respectively, one root canal in each root, with the exception of the medio-buccal root, in which a second canal occurs in 11.53-93.7% of cases [1].

The most common configuration of the mesiobuccal canals in these cases is type 2, according to Vertucci [2]. In some studies, the frequent occurrence of type 3, according to Vertucci, has also been established. Cases with more roots and canals have been reported more often in the literature. A maxillary molar with a different root canal anatomy has been reported with four roots [3], two of which may be palatal [4]. Another configuration is three buccal roots and one palatal [5].

Another variation is the fusion of the roots. In a 2020 study, Xia et al. found that fusion occurred in 31.5% of cases, with 53.2% of them having three canals and 34.9% having two canals [6]. Kim et al. found that the incidence of fused roots was 0.73% in maxillary first molars and 10.7% in maxillary second molars [7]. Upper second molars with fewer roots are less commonly reported [8-10]. Upper second molars with two roots are common (7.3%), while less common are those with one root (3.3%) [11]. A lower percentage of upper second molars with one root is observed in male patients [12]. The root canal system configurations in single-rooted upper second molars are two separate canals, one Vertucci type 1 canal and a C-shaped canal [13]. The prevalence of C-shaped root canals in maxillary second molars is about 4.5% [14].

According to Wang et al., the frequency of maxillary second molars with one root and one canal is a very rare finding (0.5-0.6%) [15,16]. In their study, Carlsen et al. analyzed the root canal system of 104 teeth with one root, mostly maxillary second molars, and found that only 25.96% of cases had one root canal located in the central part of the root [17].

In our practice, we rarely observe upper second molars with a single root canal. With this report, we present already known but important information, which through this clinical case enriches the current medical literature by sharing our experience in endodontic treatment of teeth with morphological features in the root canal system.

## Case presentation

**Figure 1:**
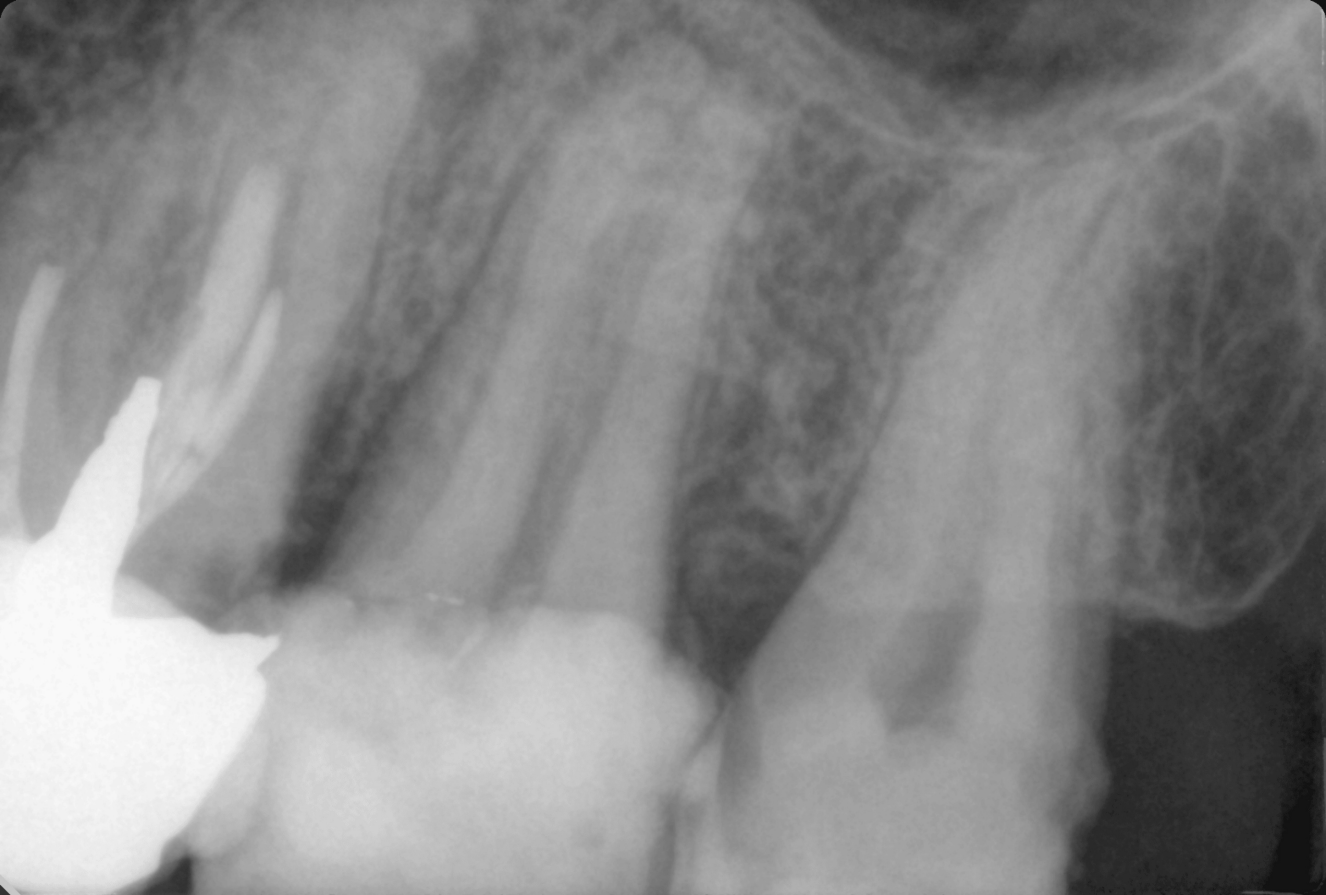
Preoperative radiograph of tooth 27

A 42-year-old man was admitted to the Medical-Dental Center at the Faculty of Dental Medicine in Varna with complaints in the area of ​​tooth 27. The patient reported pain from cold stimuli, which worsened at night. Another complaint of the patient was acute pain, which was provoked by suction. He was referred by a colleague due to the fact that he had failed to locate the pulp chamber and was worried about making an iatrogenic error. The need for additional paraclinical examination cone beam computed tomography (CBCT) was discussed with the patient, but he was not able to afford the procedure. As a result, it was decided to evaluate the case only on the intraoral periapical radiograph that the patient brought with him. After analyzing the radiograph, the vitality of tooth 27 was examined. The recorded values ​​of 58 µA confirmed the diagnosis of irreversible pulpitis of tooth 27 (Figure 1). The patient was also informed about the condition of the adjacent tooth 26, which needs to be revised, but was not the cause of the pain.

**Figure 2:**
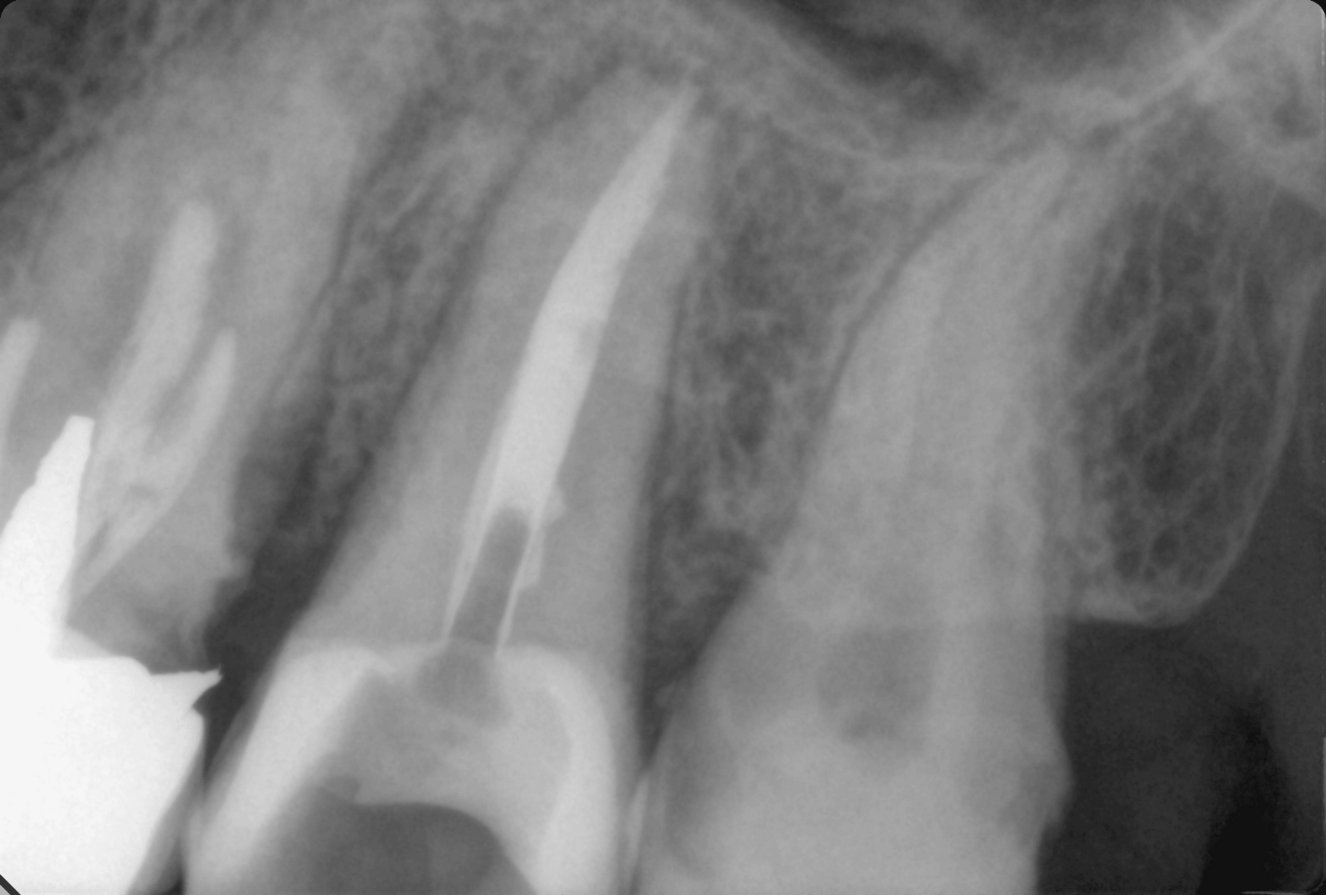
Postoperative radiograph of tooth 27

The patient did not report any concomitant diseases or medication intake. The tooth was anesthetized with 1.7 ml 4% articaine hydrochloride Septanest 40 mg/ml 1:200.000 (Septodont, Saint-Maur-des-Fossés, France). The surgical field was isolated with a rubber dam, and the removal of the temporary filling under 3.5× magnification (Univet, Rezzato, Italy) began. The residual carious mass was removed, and the pre-endodontic buildup of the medial and distal walls was performed. The pulp chamber was identified centrally within the clinical crown and was assessed using sterile Endo Z bur (Dentsply Maillefer, Woodbridge, Ontario, Canada). A wide centrally located orifice with one wide central canal was revealed. Magnification was used to carefully examine the possible existence of additional canals. The presence of only one very wide centrally located, round-shaped orifice removed any doubt about additional canals. The procedure continued with determining the working length with a K file (Dentsply Maillefer) and an apex locator FindPex (Eighteeth, Changzhou, China) 21 mm from the vestibular wall. The canal was processed with machine files from the ProTaper Next system (Dentsply Maillefer) to number X5 (50.06). Between each file, recapitulation and irrigation with sodium hypochlorite 5.25% (Cerkamed, Stalowa Wola, Poland), saline, and EDTA (Endo-Prep Gel, Cerkamed) were performed. After the final machine treatment, final irrigation was conducted, which again included 5.25% sodium hypochlorite 10 ml. It was activated using the EndoActivator (Dentsply Sirona, Charlotte, North Carolina, United States) for about 3-4 minutes. The solution was neutralized with saline rinses, followed by a 10 ml irrigation with 40% activated citric acid solution (Cerkamed). After a final saline rinse, the root canal was obturated using a single cone technique. For this purpose, calibrated gutta-percha points from the ProTaper Next system (X5) (Dentsply) and a bioceramic sealer CeraSeal (Meta Biomed, Cheongju-si, South Korea) (Figure 2) were used. After the treatment, all the patient's complaints disappeared. The tooth is due for prosthetic treatment with a zirconium crown.

## Discussion

The different configuration of a second maxillary molar with a single root canal is rare. Libfeld and Rotstein found that only 0.5% of the 200 radiographs analyzed showed this rare configuration of the root canal system [3]. A comprehensive literature search confirmed these results, showing similar results of 0.5-3.1% [18].

The variation of a second maxillary molar with a single root and a single canal is easily detected on periapical radiographs, as in this case. Conventional intraoral periapical radiographs are an important diagnostic tool in endodontics for assessing root morphology and canal configuration.

With the introduction of additional imaging studies such as CBCT into daily endodontic practice, the different types of magnification that are applied help to adequately analyze the condition of the teeth and their morphological features. Despite the advancement of technology and the wide range of additional equipment, clinical cases of maxillary second molars with a single root canal remain extremely rare. This is also confirmed by more recent studies that analyzed the morphological features of the teeth using CBCT [7].

In most cases, teeth with fewer root canals, such as the clinical case of this article, have a good prognosis due to the wide and accessible canal.

In other cases, however, if it is impossible to identify the specific configuration of the root canal system in time, the prognosis for success may deteriorate due to excessive expansion of the endodontic cavity and removal of excess dentin in the process of searching for root canals [19]. Another iatrogenic error that can be made in the process of searching for missing canals is perforation. Such iatrogenic errors can be minimized if the clinician has knowledge of the general location and dimensions of the pulp chamber. The clinician should be aware of variations in root canal anatomy, which may also occur in the form of fewer/lesser canals.

From a clinical perspective, if an atypical anatomical configuration is identified in a tooth, the symmetrical tooth should also be analyzed. An old study found that in 60% of cases, it is a symmetrical abnormal configuration of a group of teeth. They concluded that the rarer the anomaly, the more likely it is to be bilateral [20].

Morphological dental anomalies can involve a single tooth, a group of teeth, or the entire dentition [8].

## Conclusions

There are many factors that determine the successful outcome of endodontic treatment. A precise examination of the pulp chamber is mandatory and helps to localize the root canal orifices. Understanding of the anatomy of the root canal system, as well as its varieties, determines the clinical approach for each case. Knowledge of dental doctors, proper preoperative assessment, and work with magnification can be very effective in performing adequate root canal treatment in cases where a complex or rare anatomical feature is involved.
